# Editorial: Revealing the role of mitochondrial gene defects in tumor progression and developing mitochondrial-targeted drugs

**DOI:** 10.3389/fonc.2025.1553496

**Published:** 2025-02-10

**Authors:** Leilei Liang, Yuzhen Gao

**Affiliations:** ^1^ Department of Gynecological Oncology, Zhejiang Cancer Hospital, Hangzhou Institute of Medicine (HIM), Chinese Academy of Sciences, Hangzhou, Zhejiang, China; ^2^ Department of Clinical Laboratory, Sir Run Run Shaw Hospital, Zhejiang University School of Medicine, Hangzhou, Zhejiang, China

**Keywords:** mitochondrial gene defects, tumor progression, mitochondrial-targeted drugs, biomarkers, metabolism

In recent years, mitochondria have emerged as key pharmacological targets in the fight against cancer, owing to their pivotal roles in cell proliferation, apoptosis, and the tumor immune microenvironment. Mitochondrial dysfunction is increasingly recognized as a hallmark of cancer progression, with alterations in mitochondrial DNA (mtDNA), energy metabolism, and dynamics significantly contributing to tumorigenesis and therapeutic resistance (1). The concept that mitochondrial defects drive tumor progression has evolved beyond the simple notion of metabolic reprogramming (e.g., the Warburg effect), to encompass more complex mechanisms, such as mitochondrial dynamics, retrograde signaling, and mitochondrial-nuclear crosstalk (2, 3). These processes are central to the adaptation of cancer cells to the stressful microenvironment and their ability to evade therapeutic interventions. For instance, mitochondrial dysfunction can lead to the accumulation of reactive oxygen species (ROS), which further alters cellular signaling and promotes mutations (4). This Research Topic, titled “Revealing the Role of Mitochondrial Gene Defects in Tumor Progression and Developing Mitochondrial-Targeted Drugs,” offers a comprehensive and up-to-date collection of studies that provide insights into the multifaceted roles of mitochondria in cancer and highlight the potential of targeting mitochondrial dysfunction as a therapeutic strategy (5).

## 1 Mitochondria and tumor progression: from metabolic reprogramming to tumor escape

Mitochondria are essential organelles that regulate vital processes such as energy production, cell death, and stress responses. In cancer cells, mitochondrial dysfunction is often associated with altered metabolic pathways that meet the high energy demands of rapidly proliferating tumor cells (6). The reprogramming of mitochondrial metabolism is best exemplified by the Warburg effect, where even in the presence of oxygen, cancer cells predominantly rely on glycolysis for energy production, leading to the accumulation of lactate. This metabolic shift supports tumor growth and survival, but it also renders mitochondria central to the pathophysiology of cancer (7).

However, the role of mitochondria in tumor progression extends beyond metabolic alterations. Recent research has shown that changes in mitochondrial DNA copy number, mutations, and defects in respiratory chain complexes can trigger mitochondria-to-nucleus retrograde signaling (8). This process can initiate metabolic reprogramming by altering nuclear gene expression, thereby contributing to tumorigenesis and influencing the tumor microenvironment (TME). The communication between mitochondria and the nucleus thus represents a critical mechanism that governs cancer cell behavior, immune escape, and drug resistance. Studies presented in this Research Topic further support the idea that targeting mitochondrial functions—ranging from energy metabolism to mitochondrial dynamics—could disrupt these processes and provide new therapeutic opportunities for treating various malignancies.

## 2 Mitochondrial heat shock proteins: a potential target in cancer therapy

One of the pivotal articles in this Research Topic focuses on the role of mitochondrial heat shock protein 90 (mtHSP90) in cancer. Heat shock proteins (HSPs) function as molecular chaperones, helping to stabilize and refold proteins that are damaged under stressful conditions. In the context of cancer, mtHSP90 is crucial for maintaining mitochondrial integrity and the proper folding of mitochondrial proteins under stress, such as during rapid tumor growth or under conditions of hypoxia and nutrient deprivation (9). Recent studies indicate that inhibiting mtHSP90 can impair mitochondrial function and reduce tumor cell survival. The article discusses the therapeutic potential of targeting mtHSP90, highlighting preclinical evidence suggesting that mtHSP90 inhibitors could sensitize tumors to existing therapies and help overcome drug resistance.

## 3 Mitochondrial DNA mutations and biomarker development in endometrial cancer

Another critical area of exploration presented in this Research Topic is the role of mtDNA mutations in cancer, specifically in endometrial cancer. MtDNA mutations are common in various cancer types and are believed to contribute to the reprogramming of mitochondrial metabolism that supports tumor growth and survival (10). The article in this Research Topic delves into the potential use of mtDNA mutations as biomarkers for early diagnosis and prognosis in endometrial cancer. These mutations could serve as predictive markers for therapeutic response, enabling clinicians to adopt personalized treatment approaches. Moreover, understanding the role of mtDNA mutations in cancer progression could open new avenues for targeted therapies aimed at correcting mitochondrial dysfunction.

## 4 Mitochondrial permeability transition and long non-coding RNAs in clear cell renal carcinoma

A particularly intriguing study in this Research Topic investigates the role of MPT-driven necrosis-linked long non-coding RNAs (lncRNAs) in clear cell renal carcinoma (ccRCC). Mitochondrial permeability transition (MPT) refers to the opening of the mitochondrial permeability transition pore, which leads to mitochondrial swelling, rupture, and necrotic cell death. In ccRCC, MPT is implicated in promoting cell death resistance and altering the tumor microenvironment (11). The study explores how specific lncRNAs regulate MPT-driven necrosis and influence the sensitivity of ccRCC cells to chemotherapy and immunotherapy. By targeting these lncRNAs, it may be possible to enhance the effectiveness of current cancer therapies, providing a novel strategy for treating ccRCC and other cancers that exhibit MPT dysregulation.

## 5 Mitochondrial inhibitors and synergistic therapies

The Research Topic also includes a review on mitochondrial inhibitors as promising agents in cancer therapy, particularly in breast cancer. Mitochondrial inhibitors target mitochondrial metabolic pathways, such as oxidative phosphorylation, and have shown potential in preclinical studies for overcoming chemotherapy resistance and sensitizing cancer cells to treatment (12). However, clinical trials have yet to demonstrate significant benefits from mitochondrial inhibitors alone, highlighting the challenges of targeting mitochondrial functions in isolation. One emerging strategy is the synergistic use of mitochondrial inhibitors with immunotherapy, which could enhance the anti-tumor immune response and improve clinical outcomes. Combining mitochondrial inhibitors with immune checkpoint inhibitors or other immunomodulatory agents may help overcome the immune evasion mechanisms facilitated by mitochondrial dysfunction, thereby boosting the efficacy of cancer treatments.

## 6 Mitochondrial transplantation and personalized cancer therapy

In addition to direct inhibition, mitochondrial transplantation has emerged as a novel therapeutic strategy. This approach involves the transfer of healthy mitochondria into damaged or dysfunctional cells, potentially restoring normal mitochondrial function and reversing the effects of mitochondrial mutations. While this strategy is still in its infancy and faces significant technical and ethical challenges, it offers a promising direction for personalized cancer therapy, particularly for tumors driven by mitochondrial defects (13).

## 7 Future directions: a multi-omics approach

Finally, the rapid advancements in transcriptomics, single-cell sequencing, and multi-omics analysis are providing new opportunities to better understand the complex role of mitochondria in cancer. By integrating transcriptomic data with genomic, proteomic, and metabolomic profiles, researchers can identify tumor subtypes with specific mitochondrial dysfunctions and tailor therapies to target these vulnerabilities. These technologies are likely to play a crucial role in the development of mitochondrial-targeted therapies and the personalization of cancer treatment strategies (14).

## 8 Conclusion

In conclusion, this Research Topic provides valuable insights into the role of mitochondria in cancer progression and highlights the potential of mitochondrial-targeted therapies. From metabolic reprogramming to mitochondrial dynamics, mitochondria are central to tumorigenesis, immune evasion, and resistance to treatment. The studies presented in this Research Topic emphasize the need for innovative strategies that target mitochondrial dysfunction in cancer, including the development of novel mitochondrial inhibitors, the synergistic use of these inhibitors with immunotherapy, and the exploration of mitochondrial transplantation. With the continued advancement of multi-omics technologies, we are entering an exciting era of precision cancer therapy that targets the mitochondria, potentially transforming the way we treat cancer in the future.
